# Autobiographical Memory in the Angry Self

**DOI:** 10.1371/journal.pone.0151349

**Published:** 2016-03-29

**Authors:** Lynette Hung, Richard A. Bryant

**Affiliations:** University of New South Wales, Sydney, Australia; University of Akron, UNITED STATES

## Abstract

The impact of anger on autobiographical recall was examined in two studies. In Experiment 1, 76 participants differing in trait anger completed an autobiographical memory task (AMT). In Experiment 2, 50 participants with elevated trait anger were either provoked or not provoked and subsequently completed an AMT. Across both studies, participants with high dispositional anger reported more anger-related memories, describing themselves as the primary agent of anger. In Experiment 2, provoked participants reported more memories describing themselves as the target of anger. These findings highlight the distinct patterns of memory recall associated with trait versus state anger. Findings are discussed in terms of retrieval biases operating in angry individuals and proposals stemming from self-memory system models of autobiographical memory.

## Introduction

Autobiographical memories are recollections of personally experienced episodes from our past. These memories are fundamental to our sense of self, goals and motivations, and interpersonal relationships, and also allow us to make sense of the present and anticipate the future [1, 2]. Investigations of autobiographical memory have spanned a range of topics from understanding its form [1], type [3], and function [4]. Converging evidence points to the significant influence of affect on autobiographical memory. Psychopathology research suggest that disorders such as depression [5], posttraumatic stress disorder (PTSD) [6, 7], and complicated grief [8, 9] are associated with disruptions of autobiographical memory and distinct patterns of recall (for review see [3]. Interestingly, despite considerable investigation in the emotion and memory literature, a notable gap in this field involves the role of anger and personal memories. This is surprising given many contemporary accounts hold that anger follows from events in which personally significant goals are blocked by an external agent’s actions. Anger has been the focus of some studies, including those that have focused on trait anger in recalling anxious memories [10] and those that study emotion regulation strategies on anger resulting from anger-related autobiographical memories [11]. These studies have not directly investigated the impact of elicited anger on the nature of autobiographical memories. This is an important omission in the literature because studies of depression, PTSD, and grief have highlighted how other emotional states can impact on the nature of autobiographical memories is related to numerous indices of psychological functioning [12, 13].

Anger is a basic human emotion that in extreme forms can lead to a range of destructive outcomes, including verbal attacks, assault, and increased risk of marital problems and cardiovascular disease [14]. Anger can also have an adaptive function; it can enhance attention and motivation [15], increase optimism [16], and facilitate problem-solving [17]. Very few investigations have examined autobiographical memory in angry individuals. A limited amount of evidence indicates that high trait anger (HTA) is associated with negative appraisal biases for past anger-provoking events [18, 19], although HTA individuals do not differ from anxious individuals or controls in their ability to recall specific memories [19]. Notwithstanding these few findings, and in contrast to other emotional disorders, the impact of anger on autobiographical memory is unclear given the scarcity of relevant published empirical research [20].

According to the Self-Memory System Model (SMS) [1], autobiographical information is reciprocally connected to the ‘working self’, which is composed of personal goals, self-representations, and expectations. The working self modulates access to autobiographical information by facilitating and inhibiting patterns of activation (i.e., autobiographical memories), such that encoding and retrieving specific information relies on current constructions of the self. Specifically, autobiographical information that is consistent with one’s working self is more likely to be recalled than information that is inconsistent. There is empirical evidence to support this model, with studies finding that personal memories often concur with reported goals [21–23].

To extend the understanding of the role of anger on autobiographical memory, we investigated the extent to which individual differences in anger influence memory processes and whether angry individuals recall autobiographical memories in line with activated constructions of the working self. To answer these questions, we conducted two experiments that employed an adaptation of the Autobiographical Memory Task [24], during which participants recalled memories from their past in response to saliently angry, ambiguously angry, and positive cues. Experiment 1 focused on the general influence of trait anger on recalled memories. Experiment 2 built on this foundation by examining the additive impact of state anger, which was induced by interpersonal provocation, on recalled memories. Across both studies, we examined the content of recalled memories and hypothesized that anger would be associated with autobiographical memories consistent with anger themes. In this sense, we propose that anger would moderate the affective components of autobiographical memory by preferentially accessing memory representations associated with anger-related themes.

## Experiment 1

Experiment 1 investigated the influence of trait anger on autobiographical memory by comparing memories recalled by high trait anger (HTA), medium trait anger (MTA), and low trait anger (LTA) individuals. Participants were asked to generate 15 autobiographical memories in response to 5 angry, 5 ambiguously angry, and 5 positive cues; these cues were selected to determine the impact of anger on (a) salient anger cues, (b) cues intended to reflect an interpretative bias to potentially anger cues, and (c) positively valenced cues. An independent rater coded memories in terms of the degree to which they were associated with an identity that was characterized by anger. In line with proposals stemming from the SMS model, we hypothesized that HTA individuals would experience more mental representations relating being angry than LTA and MTA individuals. Accordingly, we expected HTA participants to recall more memories to angry cues in which they described themselves as the agent of anger compared to MTA and LTA participants. Specifically, the SMS model would predict that angry people would preferentially retrieve personal memories that are characterized by angry scenarios because these memories accord with their worldview that events occur to them that are anger-provoking. We also expected these memories to be associated with higher levels of anger-relatedness (i.e. mental content involving angry scenarios) than those generated by MTA and LTA participants and those recalled to ambiguously-angry and positive cues. In contrast, we expected LTA participants to recall more memories to angry cues in which they described themselves as observing others as the agent of anger emotions compared to HTA and MTA participants. Finally, we expected HTA participants to demonstrate interpretation biases to ambiguously-angry cues, in which recalled memories were associated with higher levels of anger-relatedness compared to those generated by MTA and LTA participants and those recalled to positive cues.

### Method

#### Participants

Participants were undergraduate psychology students who participated in return for course credit. Twenty-five (14 females and 11 males) HTA individuals, 26 (14 females and 12 males) MTA individuals, and 25 (14 females and 11 males) LTA individuals completed the study. Trait anger was assessed using the State-Trait Anger Expression Inventory-2: Trait Anger Scale (STAXI-2: T-Anger; [25]). Participants were identified as high, medium, or low trait anger on the basis of their STAXI-2 scores from a large initial pool of 702 participants initially screened on a 6-item adaptation of the STAXI-2: T-Anger approximately 6 months prior to the study; specifically, 25 participants were randomly identified from the top, medium, and bottom third of STAXI-2 scores. They were subsequently re-assessed on the full 10-item STAXI-2: T-Anger on average 1 week prior to the study. HTA participants scored in the range 22–33 (*M* = 26.44, *SD* = 2.86), MTA participants scored in the range 15–21 (*M* = 17.77, *SD* = 2.12), and LTA participants scored in the range 10–14 (*M* = 12.68, *SD* = 1.28) on the 10-item STAXI-2: T-Anger.

#### Materials and Measures

STAXI-2: T-Anger and STAXI-2: Anger Expression Inventory (STAXI-2: AX [25]. The STAXI-2: AX is a 32-item self-report measure consisting of two subscales that assess how individuals control their anger; the two subscales index the extent to which individuals express their anger in terms of Anger Expression-Out (e.g. “slam doors”, “say nasty things”) or Anger Expression-In (e.g. “harbour grudges”, “pout or sulk”). The scales also provide an overall anger expression index score that assesses frequency of anger expression. The STAXI-2 scales have been validated on a variety of normal and clinical populations and have good psychometric properties, with the internal consistency of the subscales ranging from 0.73 to 0.95 [25]. The STAXI-2: T-Anger is a briefer measure that comprises a 10-item self-report measure that assesses general predisposition to anger as well as predisposition to anger in specific situations. The STAXI-2: T-Anger scale correlates with other measures of trait anger, including the Buss-Perry Aggression Questionnaire (.66-.73) [26].

Anger Rumination Scale (ARS; [27]). The ARS is a 19-item self-report measure that assesses the tendency to ruminate on angry moods, past anger experiences, and the causes and consequences of anger episodes. The ARS has strong internal consistency (.93) and retest reliability (.77) [27].

State-Trait Anxiety Inventory–Form Y (STAI-Y;[28]). The STAI-Y is a 20-item self-report measure that assesses individual differences in trait anxiety, and was employed to control for trait anxiety. The STAI-Y measures level of anxiety experienced "at the moment" (state) and "generally" (trait), and possesses good internal consistency (.80) and retest reliability (.77) [28].

Beck Depression Inventory-II (BDI-II; [29]). The BDI-II is a 21-item self-report measure that assesses depressive symptomatology that has strong internal consistency of .92, and was employed to control for depressive symptoms.

Affect ratings. Participants were asked to rate the degree to which they felt happy, anxious, calm, and angry on 9-point Likert scales (*1 = not at all*, *9 = extremely*).

Positive and Negative Affect Schedule (PANAS; [30]). The PANAS comprises 20 self-reported items that index positive and negative affective states. The PANAS possesses good internal consistency (.85) and retest reliability (.71) [30]. It was used in this study to assess affective states during the experiment.

Autobiographical Memory Task (AMT). The AMT [24] consisted of five angry cues (*argue*, *furious*, *hate*, *unfair*, *violent*), five ambiguously angry cues (*chaos*, *childish*, *judge*, *jumpy*, *shout*), five positive cues (*friendly*, *merry*, *praise*, *relax*, *reliable*), and two practice cues (*movies*, *chocolate*). Test cue words were matched according to number of letters (M = 5.6–5.8), number of syllables (M = 1.6–1.8), and word frequency (M = 23.2–25.4) [31]. Cue words were initially derived from focus groups identification of explicit and ambiguous anger cue words, and then 23 postgraduate psychology students from the University of New South Wales rated the test cues for their: (1) anger-relatedness (1 = *not at all*; 9 = *extremely*), and (2) emotional valence (1 = *extremely negative*; 9 = *extremely positive*). Angry cues were rated as more anger-related than ambiguous [*t*(22) = 14.54, *p <* .001] and positive [*t*(22) = 22.46, *p <* .001] cues while ambiguous cues were rated as more anger-related than positive cues [*t*(22) = 12.83, *p <* .001]. Angry cues were also rated as more negative than ambiguous [*t*(22) = 26.22, *p <* .001] and positive [*t*(22) = 38.15, *p <* .001] cues, while ambiguous cues were rated as more negative than positive cues [*t*(22) = 20.13, *p <* .001]. Cues were presented to participants on a 20-inch computer screen using Inquisit 2.0 in 38-point Arial font in the center of the screen. Presentation order of the cues was randomized, with the constraint that positive, ambiguous, and angry cues were alternated.

Participants were instructed to verbally recall a specific autobiographical memory related to each cue. A specific memory was defined as “an event that may have lasted seconds, minutes, or even hours, but not longer than one day”, and participants were given examples of acceptable responses. Participants were given 30s to provide a memory, and responses were audio-recorded; no prompts were provided in this period.

### Procedure

Participants completed self-report measures (TAS, AXI, ARS, STAI-Y) on average one week prior to attending the experimental session. They were individually tested at the time of the experiment. This research was approved by the University of New South Wales Human Research Ethics Committee. Following written informed consent, participants completed the BDI-II and the affective rating scales to assess their current affective states. Participants then completed the AMT. At the conclusion of the AMT participants were debriefed and thanked for their participation.

### Scoring

Memory content was coded by an independent rater into three categories of self-relatedness: (1) ‘self-agent’ memories described events in which the participant was the agent or primary instigator of the event; (2) ‘self-target’ memories described events in which the participant was the target or recipient of another’s activity, and; (3) ‘other’ memories described events in which the participant was a bystander or observer to other peoples’ actions. Memory content was also coded for anger-relatedness on a 9-point Likert scale (1 = *not at all*, 9 = *extremely*). Ratings were based on the degree of anger associated with the event, the affective tone of participants’ descriptions, and participants’ tone of voice when reporting the event. A second independent rater coded 20% of participants’ responses. The mean inter-rater reliability was 0.82 (*p* < .001) for self-relatedness and .91 (*p* < .001) for anger-relatedness.

### Data Analysis

Participant characteristics were assessed between high, medium, and low trait anger groups by multiple comparisons that set a Bonferroni-adjusted alpha (*p* = .05/7 = .007). Anger-relatedness was analysed with 3 (Trait Anger) x 3 (Cue Type) mixed model analysis of covariance (ANCOVA) that controlled for depression severity (BDI) to control for the possibility that depression may account for the observed effects of trait anger. Memory content was analyzed using a 3 (Trait Anger) x 3 (Cue Type) x 3 (Content Category) multivariate analysis of variance (MANOVA), followed by separate 3 (Trait Anger) x 3 (Content Category) ANCOVAs that controlled for depression for each Cue Type.

## Results and Discussion

### Participant Characteristics

Table 1 presents mean participant characteristics. Participants did not differ in terms of age or baseline affect ratings. HTA participants scored higher than MTA participants on the STAXI-2: T-Anger [*t*(49) = 12.33, *p* < .001], AXI [*t*(49) = 4.60, *p* < .001], AX-O [*t*(49) = 6.41, *p* < .001], AC-O [*t*(49) = -3.49, *p* < .001], ARS [*t*(49) = 3.63, *p* < .001], STAI-Y [*t*(49) = 3.73, *p* < .001], and BDI-II [*t*(49) = 2.97, *p* < .01]. HTA participants also scored higher than LTA participants on the T-Anger [*t*(48) = 21.96, *p* < .001], AXI [*t*(48) = 8.37, *p* < .001], AX-O [*t*(48) = 9.28, *p* < .001], AX-I [*t*(48) = 2.50, *p* < .02], AC-O [*t*(48) = -7.78, *p* < .001], AC-I [*t*(48) = -5.04, *p* < .001], ARS [*t*(48) = 5.56, *p* < .001], and STAI-Y [*t*(48) = 5.18, *p* < .001]. MTA participants scored higher than LTA participants on the T-Anger [*t*(49) = 10.31, *p* < .001], AXI [*t*(49) = 4.27, *p* < .001], AX-O [*t*(49) = 3.47, *p* < .001], and AC-O [*t*(49) = -3.88, *p* < .001].

**Table 1:** Experiment 1: Mean participant characteristics.

	HTA (n = 25)	MTA (n = 26)	LTA(n = 25)
Age (years)	19.56 (1.98)	18.96 (1.43)	20.08 (3.24)
T-Anger	26.44 (2.86)	17.77 (2.12)	12.68 (1.28)
AX	47.28 (8.84)	35.27 (9.76)	22.12 (12.16)
AX-O	19.32 (3.22)	14.23 (2.41)	11.92 (2.34)
AX-I	18.84 (3.70)	17.54 (4.77)	15.68 (5.14)
AC-O	19.64 (3.13)	23.23 (4.12)	27.72 (4.14)
AC-I	19.12 (4.58)	21.27 (4.90)	25.76 (4.75)
ARS	39.64 (9.40)	31.65 (6.01)	27.28 (5.92)
STAI-Y	49.32 (11.59)	39.42 (6.82)	32.56 (11.31)
BDI-II	15.04 (10.32)	8.23 (5.40)	6.60 (7.72)
Baseline Affect			
Happy	6.06 (2.09)	6.40 (1.45)	6.36 (1.97)
Angry	2.81 (1.88)	2.25 (1.65)	2.00 (1.60)
Calm	6.46 (1.91)	6.02 (1.94)	5.95 (2.34)
Anxious	3.50 (1.96	2.77 (1.74)	2.45 (1.68)

Note: Standard deviations appear in parentheses. HTA = High Trait Anger; MTA = Medium Trait Anger; LTA = Low Trait Anger; TAS = Trait Anger Scale; AXI = Anger Expression Index; AX-O = Anger Expression–Out; AX-I = Anger Expression–In; AC-O = Anger Control–Out; AC-I = Anger Control–In; ARS = Anger Rumination Scale; STAI-Y = State-Trait Anxiety Inventory, Form Y; BDI-II = Beck Depression Inventory, Second Edition.

#### Memory Qualities

Table 2 presents mean anger-relatedness scores. A 3 (Trait Anger) x 3 (Cue Type) mixed model analysis of covariance (ANCOVA) that controlled for depression severity (BDI) indicated significant main effects of Trait Anger, *F*(2, 74) = 4.68, *p* < .01, *η*_*p*_^*2*^ = .11 and Cue Type, *F*(2, 73) = 80.86, *p* < .001, *η*^*2*^ = .69 but a nonsignificant interaction, *F*(2, 73) = .18, *p* >.05, *η*_*p*_^*2*^ = .04. Overall, HTA participants received higher scores than MTA [*t*(49) = 3.56, *p* < .001] and LTA participants [*t*(48) = 3.78, *p* < .001]. In contrast, MTA and LTA participants received similar scores [*t*(49) = 0.38, *p* > .71]. As expected, memories generated in response to angry cues received higher scores than memories generated in response to ambiguous [*t*(75) = 13.89, *p* < .001] and positive cues [*t*(75) = 21.65, *p* < .001]. Memories generated in response to positive cues received lower scores than memories generated in response to ambiguous cues, *t*(75) = 7.34, *p* < .001.

**Table 2:** Experiment 1: Mean anger-relatedness scores.

	HTA (n = 25)	MTA (n = 26)	LTA(n = 25)
Angry	4.72 (1.09)	3.69 (1.10)	3.53 (1.17)
Ambiguous	2.53 (1.24)	1.88 (0.88)	1.90 (0.88)
Positive	1.34 (0.59)	1.10 (0.24)	1.06 (0.18)

Note: Standard deviations appear in parentheses. HTA = High Trait Anger; MTA = Medium Trait Anger; LTA = Low Trait Anger.

#### Memory Content

Table 3 presents the mean number of memories coded into each of the three self-related content categories (self-agent, self-target, other). A 3 (Trait Anger) x 3 (Cue Type) x 3 (Content Category) multivariate analysis of variance (MANOVA) indicated a significant overall effect, *F*(18, 134) = 2.01, *p* < .05, *η*_*p*_^*2*^ = .16. Separate 3 (Trait Anger) x 3 (Content Category) ANCOVAs that controlled for depression were subsequently conducted across Cue Type. Analyses indicated that for angry cues, there was a significant main effect for Content, *F*(2, 73) = 23.55, *p* < .001, *η*^*2*^ = .39, and a significant interaction effect *F*(4, 148) = 4.15, *p* < .003, *η*_*p*_^*2*^ = .10 but a nonsignificant main effect for Trait Anger, *F*(2, 74) = 2.34, *p* >.05, *η*_*p*_^*2*^ = .06. Participants recalled more Self-Agent memories than Self-Target [*t*(77) = 8.52, *p* < .001] and Other memories [*t*(77) = 3.34, *p* < .001], and more Other memories than Self-Target memories [*t*(77) = 2.52, *p* < .02]. However, HTA participants recalled more Self-Agent memories than LTA [*t*(48) = 5.35, *p* < .001] and MTA [*t*(49) = 2.38, *p* < .02] participants. MTA participants also recalled more Self-Agent memories than LTA participants [*t*(49) = 2.98, *p* < .001]. In contrast, HTA participants recalled fewer Self-Target memories than MTA [*t*(49) = 2.03, *p* < .05] and LTA [*t*(48) = 2.94, *p* < .01]. Finally, LTA participants recalled more Other memories than HTA participants [*t*(48) = 4.37, *p* < .001], but a similar number to MTA participants [*t*(49) = 1.68, *p* > .05].

**Table 3:** Experiment 1: Mean number of self-agent, self-target, and other memories.

	HTA	HTA			MTA			MTA	
	S-Agt	S-Tar	Other	S-Agt	S-Tar	Other	S-Agt	S-Tar	Other
Angry	3.68 (1.14)	0.16 (0.37)	0.80 (0.71)	2.92 (1.13)	0.46 (0.65)	1.19 (0.94)	2.00 (1.08)	0.68 (0.80)	1.88 (1.01)
Ambiguous	2.48 (1.29)	1.00 (0.96)	1.16 (0.99)	2.19 (1.36)	0.69 (0.68)	1.73 (1.34)	2.28 (1.21)	0.88 (1.05)	1.60 (1.15)
Positive	2.56 (1.04)	1.92 (1.04)	0.20 (0.50)	2.42 (1.06)	1.77 (1.07)	0.50 (0.65)	2.36 (0.95)	1.80 (0.76)	0.56 (0.87)

Note. Standard deviations appear in parentheses. HTA = High Trait Anger; MTA = Medium Trait Anger; LTA = Low Trait Anger; S-Agt = Self-Agent; S-Tar = Self-Target.

Note: Standard deviations appear in parentheses. HTA = High Trait Anger; MTA = Medium Trait Anger; LTA = Low Trait Anger; S-Agt = Self-Agent; S-Tar = Self-Target.

For ambiguous cues, there was a significant main effect for Content [*F*(2, 73) = 7.04, *p* < .002, *η*_*p*_^*2*^ = .16]. Participants recalled more Self-Agent memories than Self-Target [*t*(370) = 5.80, *p* < .001] and Other memories [*t*(370) = 3.03, *p* < .01], and more Other memories than Self-Target memories [*t*(370) = 3.22, *p* < .001]. For positive cues, there was a significant main effect for Content [(2, 73) = 56.88, *p* < .001, *η*_*p*_^*2*^ = .61]. Participants recalled more Self-Agent memories than Self-Target [*t*(77) = 4.26, *p* < .001] and Other [*t*(77) = 7.73, *p* < .001] memories, and more Self-Target memories than Other memories [*t*(77) = 2.90, *p* < .005].

These findings indicate an autobiographical memory pattern associated with trait anger. Specifically, HTA appears to be strongly associated with a tendency to recall angry memories that are thematically aligned with views of the self as an ‘angry’ individual; that is, as someone who experiences anger-related emotions and behaviors. These findings are consistent with the Self-Memory System Model proposition that an individual’s working self-image will shape the nature of their autobiographical recall. We next considered the impact of manipulating state anger and correspondingly, individuals’ self-image, on autobiographical memory in Experiment 2.

## Experiment 2

Experiment 2 investigated the impact of state anger on autobiographical memory by comparing the memories recalled by angry and non-angry participants. Previous research has used a range of paradigms to elicit anger in the laboratory [32, 33]. One widely used technique involves interpersonal provocation in which participants are asked to perform a task, during or following which they are deliberately insulted by the experimenter or a confederate [34]. In Experiment 2 medium-high trait anger participants were either provoked or not provoked in order to induce anger in half the participants; participants then completed the AMT. On the basis of the SMS model, we propose that the interpersonal nature of the provocation would prompt provoked individuals to have more highly activated self-representations related to being victims of goal obstruction and injustice than non-provoked participants. Accordingly, we expected non-provoked participants to behave similarly to HTA individuals in Experiment 1 and recall more memories to angry cues in which they described themselves as the agent of anger-related emotions. Further, we expected provoked participants to recall more memories to angry cues in which they described themselves as targets of injustice and insult by others. Finally, we expected participants to demonstrate interpretation biases to ambiguously-angry cues: it was predicted that non-provoked participants would recall more memories in which they were the agent of the emotional experience, whereas provoked participants would recall more memories in which they were the target of the semotional experience. Memories recalled to ambiguous cues from both groups were expected to be associated with higher levels of anger-relatedness compared to those recalled to positive cues.

## Method

### Participants and Design

Participants were undergraduate psychology students who participated in exchange for course credit. Fifty (42 females and 8 males) individuals were randomly assigned to receive either a provocation or no provocation induction. Participants were selected from a large initial pool of 355 individuals first screened on a 6-item adaptation of the STAXI-2: T-Anger scale approximately 5 months prior to the study and were subsequently re-assessed on the full 10-item version of the STAXI-2: T-Anger one week prior to the study. Participants scored in the medium-high range 15–38 (*M* = 21.56, *SD* = 6.03) on the full version of the STAXI-2: T-Anger.

### Measures

Participants completed the STAXI-2: T-Anger, STAXI-2: AX, ARS, STAI-Y, and BDI-II (see Experiment 1). Participants also completed the PANAS-X, a widely used 24-item self-report measure that assesses state affect at given points in time.

Participants completed a 15-item list of anagrams [34]. Provoked participants completed a set comprising difficult anagrams (e.g., dmmpaiunneo = pandemonium). Non-Provoked participants completed a set comprising easy anagrams (e.g., rfsto = frost). All participants were given 4 minutes to complete the anagram task. The AMT was identical to Experiment 1.

### Procedure

Participants completed self-report personality measures (STAXI-2: T-Anger, STAXI-2: AX, ARS, STAI-Y) one week prior to attending the experimental session. They were individually tested at the time of the experiment, and their responses were filmed. On arrival in the laboratory, they were told that the study was investigating the relationship between cognitive abilities and autobiographical memory. This study was approved by the UNSW Human Research Ethics Committee. Following written informed consent, participants completed the PANAS-X and the BDI-II to assess their current affective states. They were then asked to complete a set of anagrams (the difficulty of which depended on their allocated group) as a measure of their cognitive ability. The experimenter left the laboratory for three-minutes to score participants’ performance; responses were marked using either a red pen (provocation group) or green pen (no-provocation group). Upon her return, irrespective of their actual performance on the anagram task, she presented participants with feedback regarding their scores. Provoked participants were insulted in an irritated and exasperated tone of voice: ‘‘You really got a lot of these wrong. This data is useless to me. We should probably just start all over, but to be perfectly honest with you, I don’t want to waste my time.” Non-Provoked participants were told in a neutral tone of voice: “Your performance on this task was fine. We can use this data. In fact, you performed within the average range. This is generally what we’ve found from other people around your age at university.”

Following feedback, participants were lead through instructions for the AMT and practice trials. The experimenter then told Provoked participants, again in an irritated tone of voice: “Let’s see if you can do better on this task than you did on the anagram task.” She told Non-Provoked participants in a neutral tone: “You’re performing fine so far. You seem to know what to do.” Participants then proceeded to complete the AMT. At the conclusion of the AMT, participants completed a second PANAS-X which was modified to assess their affective reactions at the time immediately following the provocation. Participants were also asked to rate the extent to which they thought about their anagram performance during completion of the AMT (1 = “I didn’t think about it at all”; 9 = “I thought about it the whole time”). They were then debriefed and thanked for their participation.

### Scoring

Memory content was coded in an identical manner to Experiment 1. A second independent rater coded 25% of participants’ responses. The mean inter-rater reliability was 0.88 (*p* < .001) for self-relatedness and 0.93 (*p* < .001) for anger-relatedness.

Facial expressions prior to and during anagram feedback were scored from video-recordings of participants by a rater who was blind to participants’ experimental condition. Scoring was based on the Facial Action Coding System (FACS; [35], which is a system used to categorize facial muscle movement or ‘facial action units’ (FAUs) in the expression of emotions. For the purposes of this study, FAUs specific to the expression of anger were identified, and included: (a) inner brow lowerer, (b) upper lip raiser, (c) lid tightener, and (d) lip tightener. Participants were scored on the degree to which they displayed movement in these FAUs using an 8-point Likert-scale (0 = *no movement*, 7 = *extreme movement*). Participants were additionally scored on the global intensity of expression using an 8-point Likert scale (1 = *not at all angry*, 8 = *extremely angry*). An independent rater coded 25% of facial expressions. The mean inter-rater reliability was 0.90 (*p* < .001) for the four FAUs and 0.87 (*p* < .001) for global intensity.

### Data Analysis

Participant characteristics were assessed between induction conditions by multiple comparisons that set a Bonferroni-adjusted alpha (*p* = .05/9 = .006). Anger-relatedness was analysed with 2 (Provocation Group) x (3) (Cue Type) mixed model ANCOVA that controlled for depression severity. Memory content was analyzed using a 2 (Provocation Group) x 3 (Cue Type) x 2 (Content Category) multivariate analysis of variance (MANOVA) indicated a significant overall effect [*F*(6, 43) = 2.32, *p* < .05, *η*_*p*_^*2*^ = .24], followed by separate. 2 (Provocation Group) x 3 (Content) ANOCVAs.

## Results and Discussion

### Participant Characteristics

Table 4 presents mean participant characteristics. There were no differences between groups in terms of participants’ age, or scores on the STAXI-2: T-Anger, STAXI-2: AXI, AX-O, AC-O, AC-I, ARS, STAI-Y, or BDI-II scores.

**Table 4:** Experiment 2: Mean participant characteristics.

	Provoked (n = 25)	Non-Provoked (n = 25)
Age (years)	20.04 (4.35)	19.60 (2.80)
T-Anger	21.88 (5.99)	21.24 (6.18)
AXI	44.72 (11.69)	43.92 (11.00)
AX-O	19.16 (4.05)	18.88 (3.76)
AX-I	18.92 (3.98)	16.12 (4.14)
AC-O	21.44 (3.81)	20.04 (3.94)
AC-I	19.92 (4.54)	19.04 (4.58)
ARS	40.32 (10.62)	36.64 (11.35)
STAI-Y	44.20 (9.02)	44.24 (9.80)
BDI-II	10.64 (5.61)	10.12 (7.59)

Note: Standard deviations appear in parentheses. T-Anger = Trait Anger Scale; AXI = Anger Expression Index; AX-O = Anger Expression–Out; AX-I = Anger Expression–In; AC-O = Anger Control–Out; AC-I = Anger Control–In; ARS = Anger Rumination Scale; STAI-Y = State-Trait Anxiety Inventory, Form Y; BDI-II = Beck Depression Inventory, Second Edition.

### Manipulation Checks

Table 5 presents participants’ ratings on the PANAS hostility subscale pre- and post-anagram feedback. A 2 (Provocation Group) x (2) (Time) mixed model ANOVA indicated a significant interaction effect [*F*(1, 47) = 16.47, *p* < .001, *η*_*p*_^*2*^ = .03]. Provoked participants scored higher on the hostility scale following anagram feedback compared to baseline. In contrast, Non-Provoked participants scored lower on the hostility scale following anagram feedback compared to baseline. Table 5 also presents facial expression scores following anagram feedback. Provoked participants demonstrated significantly more anger-related eye movements than Non-Provoked participants, [*t*(46) = 2.62, *p* < .03]. There were no differences between the two groups on anger-related eyebrow [*t*(46) = 0.43, *p* >.05] or mouth movement scores [*t*(46) = 1.02, *p* >.05]. Collectively, in view of PANAS and facial expression scores, it appears that the provocation manipulation successfully induced angry affect in Provoked participants.

**Table 5:** Experiment 2: Mean PANAS ratings, facial expression scores, and anger-relatedness scores.

	Provoked	Non-Provoked
PANAS–Hostility		
Pre-Feedback	1.24 (0.39)	1.50 (0.77)
Post-Feedback	1.62 (0.74)	1.29 (0.51)
Facial Expression		
Eyebrows	1.10 (0.45)	1.17 (0.57)
Eyes	1.71 (0.91)	1.21 (0.59)
Mouth	3.63 (1.61)	3.13 (1.77)
Anger-Relatedness		
Angry	5.61 (1.01)	5.08 (0.90)
Ambiguous	2.66 (0.88)	2.45 (0.78)
Positive	1.34 (0.30)	1.36 (0.63)

Note: Standard deviations appear in parentheses.

### Anger-Relatedness

Table 5 presents mean anger-relatedness scores. A 2 (Provocation Group) x (3) (Cue Type) mixed model ANCOVA that controlled for depression indicated a significant main effect of Cue Type [*F*(2, 46) = 335.95, *p* < .001, *η*_*p*_^*2*^ = .94] but a nonsignificant main effect for Provocation Group [*F*(1, 47) = 2.72, *p* >.05, *η*_*p*_^*2*^ = .05] and a nonsignificant interaction [*F*(2, 46) = 2.07, *p* >.05, *η*_*p*_^*2*^ = .08]. As expected, memories generated in response to angry cues received higher anger-relatedness scores than memories generated in response to ambiguous [*t*(49) = 20.38, *p* < .001] and positive cues [*t*(49) = 29.28, *p* < .001]. Memories generated in response to positive cues received lower scores than memories generated in response to ambiguous cues [*t*(49) = 9.90, *p* < .001].

### Memory Content

Table 6 presents the mean number of memories coded into each of the three self-related content categories (self-agent, self-target, other). A 2 (Provocation Group) x 3 (Cue Type) x 2 (Content Category) multivariate analysis of variance (MANOVA) indicated a significant overall effect [*F*(6, 43) = 2.32, *p* < .05, *η*_*p*_^*2*^ = .24]. Separate 2 (Provocation Group) x 3 (Content) ANOCVAs that controlled for depression were subsequently conducted across angry, ambiguous, and positive cues. Analyses indicated that for angry cues, there was a significant main effect for Content, [*F*(2, 46) = 13.44, *p* < .001, *η*_*p*_^*2*^ = .37], and a significant interaction effect [*F*(2, 46) = 5.12, *p* < .01, *η*_*p*_^*2*^ = .18] but a nonsignificant main effect for Provocation Group [*F*(1, 47) = 2.02, *p* >.05, *η*_*p*_^*2*^ = .001]. Participants recalled more Self-Agent memories than Self-Target [*t*(49) = 6.14, *p* < .001] and Other [*t*(49) = 3.57, *p* < .001] memories, and more Other memories than Self-Target memories [*t*(49) = 2.59, *p* < .01]. Whereas Non-Provoked participants recalled more Self-Agent memories than Provoked participants [*t*(48) = 2.65, *p* < .01], Provoked participants recalled more Self-Target memories than Non-Provoked participants [*t*(48) = 2.56, *p* < .01]. For ambiguous cues, there was a significant main effect for Content [*F*(2, 46) = 25.38, *p* < .001, *η*_*p*_^*2*^ = .53], but a nonsignificant effect for Provocation Group [*F*(1, 47) = 2.00, *p* >.05, *η*_*p*_^*2*^ = .04] and the interaction [*F*(2, 46) = 2.36, *p* >.05, *η*_*p*_^*2*^ = .09]. Participants recalled more Self-Agent memories than Self-Target [*t*(49) = 6.82, *p* < .001] and Other memories [*t*(49) = 2.30, *p* < .02], and more Other memories than Self-Target memories [t(49) = 4.60, *p* < .001]. For positive cues, there was a significant main effect for Content [*F*(2, 46) = 137.94, *p* < .001, *η*_*p*_^*2*^ = .86] but a nonsignificant main effect for Provocation Group [*F*(1, 47) = 1.96, *p* >.05, *η*_*p*_^*2*^ = .04] and a nonsignificant interaction [*F*(2, 46) = 0.33, *p* >.05, *η*_*p*_^*2*^ = .01]. Participants recalled more Self-Agent memories than Self-Target [*t*(49) = 3.47, *p* < .001] and Other [*t*(49) = 15.88, *p* < .001] memories, and more Self-Target memories than Other memories [*t*(49) = 11.26, *p* < .001].

**Table 6:** Experiment 2: Mean number of self-agent, self-target, and other memories.

	Provoked			Non-Provoked		
	S-Agent	S-Target	Other	S-Agent	S-Target	Other
Angry	1.96 (1.10)	1.12 (0.97)	1.36 (1.04)	2.84 (1.25)	0.52 (0.65)	1.36 (1.08)
Ambiguous	2.00 (1.29)	0.76 (0.83)	1.92 (1.22)	2.84 (1.21)	0.60 (0.82)	1.44 (1.04)
Positive	2.56 (0.77)	1.92 (0.81)	0.28 (0.54)	2.76 (0.93)	1.92 (0.76)	0.24 (0.44)

Note: Standard deviations appear in parentheses. S-Agent = Self-Agent; S-Target = Self-Target.

Finally, there were also no differences between groups in terms of the extent to which they thought about their performance on the anagram task during the AMT, t(42) = 1.19, p>.24.

These findings provide initial evidence of an autobiographical memory pattern associated with state anger that was independent of mood congruency, emotionality and affective tone effects. Specifically, anger induced via interpersonal provocation appears to be associated with the tendency to recall angry memories that are thematically and emotionally aligned with views of the self as the target of anger and injustice. In contrast, as in Experiment 1, in the absence of interpersonal provocation, angry memories are more likely to be aligned with views of the self as the agent of anger-related emotion. Both these findings are consistent with the Self-Memory System Model’s proposition that an individual’s current, activated working self-image will shape the nature of their autobiographical recall. Moreover, findings partly indicate that when provoked, participants are less likely to recall ambiguously-angry memories associated with views of the self as the agent of anger, which may indicate increased sensitivity biases to ambiguous information.

## General Discussion

Collectively, we observed reliable effects of HTA on the nature of recalled memories. Specifically, HTA participants in Experiment 1 recalled more angry memories in which they described themselves as the primary agent of anger and related emotions compared to MTA and LTA participants. These participants were mimicked by Non-Provoked HTA (i.e., control) participants in Experiment 2, who also recalled more angry memories in which they were the agent of anger and related emotions compared to Provoked HTA participants. These findings are in line with our proposition that HTA may generally be associated with views of oneself as an angry person, and that HTA individuals will generate memories consistent with this self-perception. These findings are also consistent with evidence from the broader psychopathology literature that individuals with complicated grief and PTSD tend to recall memories consistent with their self-image [21, 23]. Thus, in terms of the Self-Memory System Model, trait anger may have constrained the accessibility and retrieval of particular autobiographical information via differentially activating self-representations associated with personal anger proneness and reactivity; this interpretation is speculative because we did not directly index self-representations.

Provoked HTA participants in Experiment 2 displayed noticeable differences in their patterns of recall compared to Non-Provoked participants: they recalled more angry memories in which they were the primary target of anger and injustice and fewer memories to ambiguously-angry cues in which they were the primary agent of anger. These findings indicate that elevated state anger can significantly alter the nature of HTA individuals’ autobiographical memories. Specifically, these findings are in line with our proposition that when angered (via interpersonal provocation), HTA individuals have activated views of themselves as the subject of injustice, and accordingly retrieve autobiographical information consistent with this image (or ignore information conflicting with this self-image). More generally, these findings suggest that manipulating state affect may alter goals, representations, and images associated with the working self, which in turn impacts the construction and retrieval of autobiographical information.

An individual’s autobiographical knowledge base contains a range of negative, anger-related experiences (as well as positive and neutral experiences). The findings from Experiments 1 and 2 distinguish between the impact of anger as stable, personality construct and anger as a transient state on retrieval of information from the autobiographical knowledge base. The pattern of findings across the two studies provides strong support for the notion that accessibility of anger-related autobiographical memories, which is closely aligned with the working self, is differentially influenced by trait and state anger factors.

It is important to recognize that Provoked HTA participants in Experiment 2 demonstrated elevated levels of state anger in a specific context; anger representations were activated and retrieval searches were guided via an interaction that was highly salient, provocative, and interpersonal [36] [37]. In light of these circumstances, changes to state affect and the working self seemed to facilitate the recall of memories that promoted, and perhaps even reinforced, images of the self as targets (rather than agents) of anger and injustice. Thus, it is possible that changes in the working self that occur when state anger is induced—but in a context free of interpersonal provocation (e.g., via exposure to anger-inducing material [38])—may motivate alternate patterns in the content of recalled autobiographical memories.

Our findings shed new light on how autobiographical memory may function in angry individuals and the role of the dynamic working self in the retrieval of anger-related autobiographical information. We note that we cannot exclude the possibility that the results may be influenced by mood congruency effects [39]. These studies did not closely assess emotional reactivity in association with changing anger levels. Future studies should consider the role of anger in the context of other emotional states to isolate the effect of anger on autobiographical memory from other emotional states.

Interestingly, our findings revealed evidence for cue-specific effects. Overall, irrespective of trait or state factors, participants across both studies displayed similar patterns in the content of memories generated in response to positive cues; they recalled more memories in which they were the primary agent, rather than target, of positive action. There were some differences in the content of memories generated in response to ambiguously-angry cues, but these were restricted to Provoked participants in Experiment 2 who reported events in which they were less likely to be the agent. The predominant differences in content–both within and across the two studies–were found for memories generated in response to angry cues: whereas in the absence of provocation (Experiment 1) HTA participants generated memories which described themselves as the primary instigator of anger, following provocation (Experiment 2) they described themselves more so as the target or recipient of anger. Collectively, these findings suggest that both trait and state anger are associated with an underlying retrieval search bias specific to angry memories and also, to a lesser extent, ambiguously-angry memories. This memory search bias may be related to other cognitive processes associated with HTA. In particular, these findings are consistent with evidence indicating that HTA individuals display selective attention biases to anger-related stimuli in the absence of provocation [40, 41] as well as following provocation [42–44]. These findings are also in line with evidence highlighting that HTA individuals display reasoning biases to ambiguously hostile information [45, 46]. Taking into account the distinct patterns in the content of recalled memories across the two studies, this search bias may also emerge from current activations in an individual’s self-image.

These findings have implications for understanding the way in which anger shapes the working self and subsequently, the way in which the self interacts with autobiographical information to propagate or maintain anger symptoms. General anger-proneness or activated views of oneself as a victim of injustice and the retrieval of memories consistent with either or both of these images may contribute to ongoing emotional reactivity, and even increased vigilance to anger-provoking stimuli, when confronted by potential threat and injustice. Relatedly, the cyclical interaction between anger, the self and autobiographical memory may reduce an angry individual’s ability to exert effortful control (i.e., inhibit dominant responses, detect errors, and engage in planning [47]) in order to regulate anger [48]. In turn, these factors may contribute to the maintenance of anger reactions.

We acknowledge limitations of the present research. First, it is possible that the mood induction used in Experiment 2 promoted a ruminative response, which in turn may have influenced the content of recalled memories. We did not index rumination in relation to the specific experimental tasks, including the provocation procedure, and so we cannot conclude the role that rumination may have played in the observed retrieval patterns. In light of evidence that rumination impacts on autobiographical memories [49, 50], future research should study the role of rumination following anger inductions and the impact on autobiographical memory. Second, we did not measure physiological arousal, which is a typical feature of state anger [51]. Future studies should experimentally manipulate arousal levels to index the impact of physiological arousal because this can impact on executive control capacity, which in turn influences autobiographical retrieval [52]. Third, although the mood induction was successfully administered, social demands associated with the university experimental setting (e.g., university students participating in exchange for course credit) may have limited the degree to which participants experienced/expressed anger. We were not able to accurately index the extent to which participants were aware of the intent of the anger provocation, which may have influenced their responses. Future studies are needed to examine the generalizability of these findings to community and clinical populations. Fourth, it is possible that numerous other factors may have driven the observed effects, such as sex, depression, attitudinal factors, or sociodemogrpahic variables; future studies need to be adequately powered and use suitable measures to control for these variables. Fifth, we did not include a LTA control group in Experiment 2. Finally, we did not index the full range of facial responses according to the Ekman scoring system for facial emotion expression [35]; this may have provided a more accurate index of anger expression.

These findings point to the critical role of anger in autobiographical memory. They provide novel evidence indicating that distinctive patterns of autobiographical memory are associated with anger at the trait versus state levels. Inducing anger, and in so doing, temporarily altering the working self, led to important differences in content of individuals’ retrieved memories. This suggests that concentrated efforts to change one’s experiences of anger (maladaptive) self-image may produce powerful effects in memory retrieval and anger symptoms. Developing the research base of the role of autobiographical memory associated with anger has the potential to markedly enhance the current models of anger.

## Supporting Information

S1 Study 1 Data (SAV)

S2 Study 2 Data (SAV)
